# Effectiveness and safety of intraoperative intraperitoneal 5-Fu drug implantation in patients with colorectal cancer: a retrospective cohort study

**DOI:** 10.1007/s00432-023-05523-2

**Published:** 2024-02-13

**Authors:** Renchao Liu, Xianqin Hu, Chen Lai

**Affiliations:** 1 Department of General Surgery, Xiangya Hospital, Central South University, Changsha, Hunan People’s Republic of China; 2 National Clinical Research Center for Geriatric Disorders, Xiangya Hospital, Central South University, No. 87, Xiangya Road, Changsha, 410008 Hunan China; 3 Hunan Key Laboratory of Precise Diagnosis and Treatment of Gastrointestinal Tumor, Xiangya Hospital Central South University, Changsha, Hunan China; 4 International Joint Research Center of Minimally Invasive Endoscopic Technology Equipment & Standardization, Changsha, China

**Keywords:** Sustained-release 5-fluorouracil, Colorectal cancer, Safety, Long-term effect, Postoperative chemotherapy

## Abstract

**Purpose:**

The purpose of this clinical study was to evaluate the efficacy and safety of intraoperative chemotherapy (IOC) with intraoperative intraperitoneal implantation of 5-fluorouracil (5-FU) in colorectal cancer (CRC) patients.

**Methods:**

In this study, 165 patients who underwent colorectal radical surgery were selected, of whom 111 in the experimental group received surgical treatment with an intraperitoneal 5-fluorouracil (5-FU) implantation. Fifty-four patients who did not undergo intraperitoneal implantation of 5-FU were matched to compare the progression-free survival (PFS) and overall survival (OS) with the former.

**Results:**

We also studied the differences in the changes of different biochemical indicators between the two groups before and after surgery, and there were significant differences in leukocytes, neutrophils, and lymphocytes before and after (*P* < 0.05), while for sodium ions, potassium ions, platelets, alanine transaminase, aspartate transaminase, creatinine, urea, and albumin, there were no significant differences. This may be related to the intraperitoneal chemotherapy implant entering the blood circulation. For 5-year OS, there were 85/111 (76.58%) in the 5-FU group (*P* = 0.013) and 35/54 (64.81%) in the control group; for 5-year PFS, there were 84/111 (75.68%) in the 5-FU group and 29/54 (53.70%) in the control group (*P* = 0.02). All the experimental groups were better than the control group with a significant difference in the experimental results.

**Conclusion:**

For CRC surgery patients, intraperitoneal implantation of slow-release 5-FU drugs, which is a safe and simple procedure, can improve the prognosis of the patients.

**Clinical trial registration:**

No clinical trials were performed in the study.

## Introduction

Colorectal cancer (CRC) is the third most common malignancy worldwide and the second most deadly malignancy (Sung et al. 2021). The current treatment option for resectable colorectal cancer is colorectal resection supplemented by adjuvant chemotherapy. NCCN guidelines recommend adjuvant chemotherapy for stage II patients with high-risk factors and stage III or IV patients, which can effectively improve their prognosis. 5-Fluorouracil (5-FU) is an important component of systemic chemotherapy for CRC in both palliative and adjuvant settings. The common chemotherapy regimens for CRC include Cape, Capeox, FOLFOX, etc. For patients who are unable to receive combination chemotherapy, the NCCN continues to advocate oral 5-FU-based agents such as capecitabine single-agent chemotherapy. Chemotherapy regimens with 5-FU as the core drug can be effective in improving patients’ outcomes. However, as studies have shown, approximately one-third of colorectal patients experience recurrence after radical surgery (Harris et al. 2002). How to improve the survival and reduce the recurrence of this subset of patients is becoming an urgent issue.

Although systemic chemotherapy involving 5-FU remains the cornerstone of treatment for CRC patients as we enter the era of personalized cancer drugs and new anti-tumor agents and treatment principles continue to evolve (Vodenkova et al. 2020), 5-FU has a short half-life and is rapidly eliminated after intravenous administration into the circulation (Sun et al. 2017). For intraperitoneal drug perfusion, it is important to maintain a more stable drug concentration. Increasing the intravenous drug concentration is not effective in maintaining the intraperitoneal drug concentration and is accompanied by non-negligible side effects. In recent years, we have witnessed the development of related sustained-release drugs, which supported maintaining intraperitoneal drug concentration for a long time. As one of the examples of sustained-release formulations, implant 5-FU sustained-release formulations prolong the duration of the drug’s activity in the body, enhance safety, and optimize the drug’s absorption. The results indicate that 5-FU implant is more effective and safer than 5-FU bolus injection, and could also be used to conquer the recurrence of postoperative colon cancer (Li et al. 2018).

Our present clinical study aims to evaluate the efficacy and safety of intraoperative chemotherapy (IOC) with flunosamine administered by intraoperative intraperitoneal implantation in patients with CRC in the hope of discovering an effective and usable method to address the residual malignant cells in the peritoneal cavity resulting from surgery. This is a safe and simple procedure that might increase patient benefit.

## Patients and method

### Patients

**Fig. 1:**
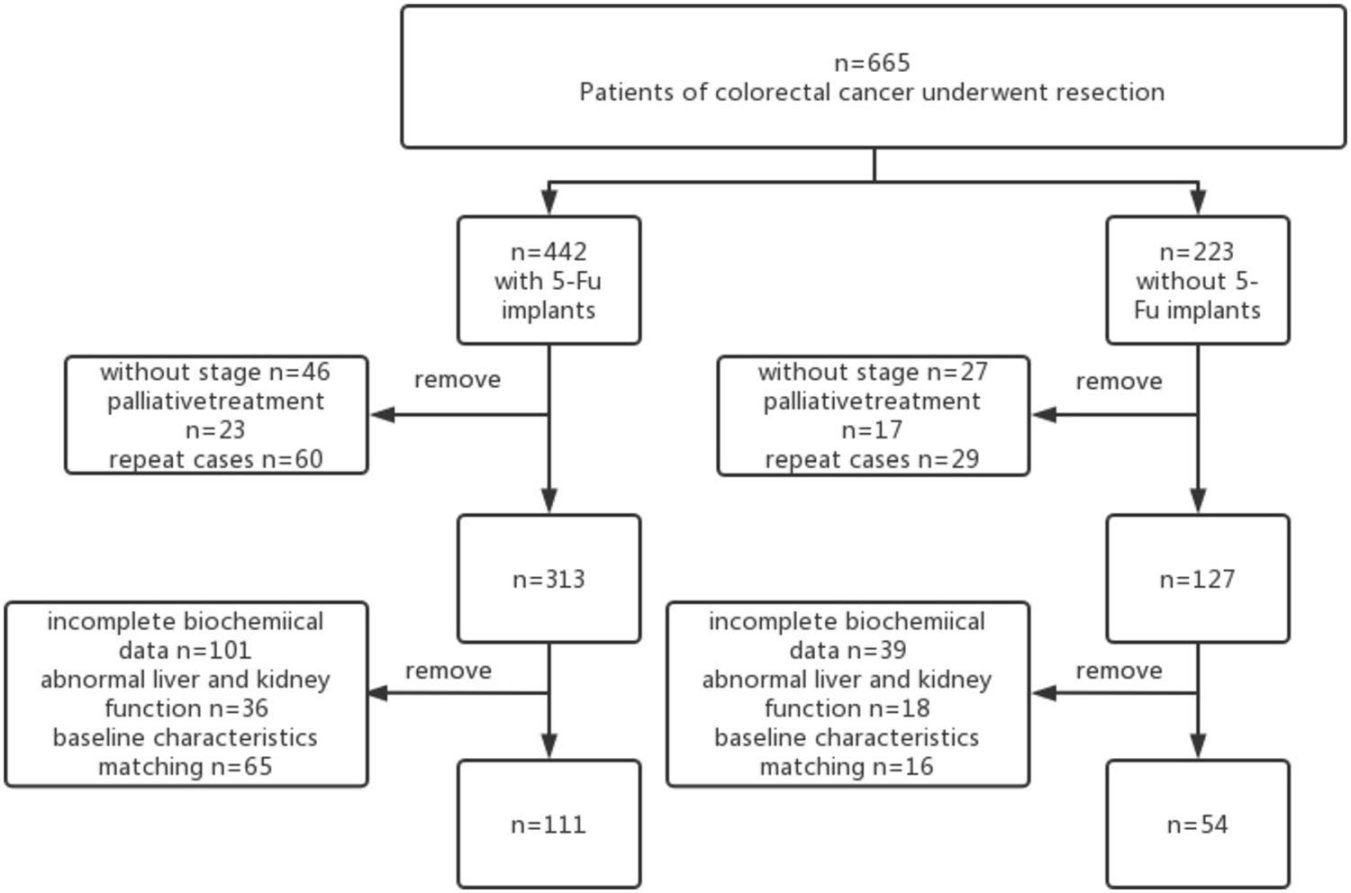
flowchart of the selection of included patients. 5-FU,5-fluorouracil

A total of 165 patients who underwent colorectal radical surgery at Xiangya Hospital of Central South University, Changsha, Hunan Province, China, between January 2011 and June 2017 were included in this study. Among them, the experimental group had 111 patients who underwent standard surgical treatment with intraperitoneal implantation of 5-FU matched with another 54 patients who did not receive intraperitoneal implantation of 5-FU based on the baseline characteristics of these 111 patients. The flow of the included participants in the study is shown in Fig. 1. And there was no significant difference in the population characteristics between the two groups. We compared the clinical characteristics, short-term postoperative prognosis, safety, progression-free survival (PFS), and overall survival (OS) between the above two groups. The inclusion criteria were: (1) laparoscopic or open radical surgery in Xiangya Hospital 2011–2017; (2) colorectal cancer confirmed by pathological examination; 3) without neoadjuvant chemotherapy, and colorectal cancer was the only primary carcinoma; (4) data integrity. Exclusion criteria were: (1) palliative care; (2) hepatic and renal dysfunction, severe neuropathy or psychiatric illness; and (3) having two or more primary malignancies of different organ and tissue origin; (4) chemotherapy is not considered appropriate by the investigator. This study was approved by the ethics committee of Xiangya Hospital, Central South University—ethical approval number: 202201013.

### Experimental design

#### Sustained-release 5-FU administration

In 5-FU implants group, a total dose of 600–800 mg 5-FU implants (Sinofuan®, Wuhu Zhongren Pharmaceutical Co.,Ltd. Anhui Province, China) was dispersed in the tumor bed, pelvic cavity, paracolonic groove, and subdiaphragm at the end of surgery. The dosage of drug was calculated by patients’ body surface area, i.e., the dosage was 400 mg/m^2^. It is worth noting that 5- FU implants were always placed in a standard position to avoid direct contact of the implant with skeletal, vessels, and anastomoses. Meanwhile, the surface of the small intestine, anastomosis, and exposed vessels were carefully avoided. In China, the first sustained-release anti-tumor implant product approved by the CFDA was a polymer carrier containing 5-fluorouracil components named Sinofuan (Medicines NO. H20030345, Patent No. 1028685). Sinofuan is an analog of uracil, a component of ribonucleic acid. The drug is believed to function as an antimetabolite. After intracellular conversion to the active deoxynucleotide, it interferes with the synthesis of DNA by blocking the conversion of deoxyuridylic acid to thymidylic acid by the cellular enzyme thymidylate synthetase. Sinofuan may also interfere with RNA synthesis. After intravenous administration, Sinofuan is distributed through the body water and disappears from the blood within 3 h. It is preferentially taken up by actively dividing tissues and tumors after conversion to its nucleotide. Sinofuan readily enters the C.S.F. and brain tissue. Following I.V. administration, the plasma elimination half-life averages about 16 min and is dose dependent. Following a single I.V. dose of Sinofuan, approximately 15% of the dose is excreted unchanged in the urine within 6 h; over 90% of this is excreted in the first hour. The remainder is mostly metabolized in the liver by the usual body mechanisms for uracil.

The operating surgeon performed colorectal cancer resection in strict accordance with standard surgical procedure, and after completion of the operation, mesonephric fluralaner was encapsulated using absorbable material and placed separately in the tumor bed, the root of the mesocolon, and adjacent to the paracolic gutter, taking care to avoid the anastomosis and the wall of large blood vessels.

### Safety evaluation

The safety evaluation assessed the effect of the treatment on recovery, abdominal cavity, and other organ functions according to common toxicity criteria released by the National Cancer Institute (nci-ctcae v4.0). Fever, pulmonary infection, anastomotic leak, and other complications were recorded. In addition, other parameters included gastrointestinal toxicity (including vomiting, diarrhea, and bleeding), hematologic toxicity (including white blood cell count, red blood cell count, and platelet count before and 7 days after surgery), renal toxicity (including elevation of blood urea nitrogen (urea nitrogen) and creatinine), and hepatotoxicity (including elevation of enzymes such as alanine aminotransferase).

### Statistics

SPSS 22.0 and The GraphPad Prism 8 software were used for statistical analysis, and clinical trial results were presented as mean ± SD. Quantitative indicators were described by means, standard deviation, median, maximum, and minimum values. Intergroup comparison was conducted using the log-rank test. Variables were described by number and percentages; Chi-square test was used for within-group comparisons of categorical variables. All statistical tests were two-sided. Wilcox rank sum test and paired *t* test were performed to investigate the hematologic toxicity within and between the groups to evaluate whether 5-FU implants would cause additional adverse reactions. Complications and adverse effects were evaluated by group *t* test. In addition, Bonferroni post hoc analysis was performed for multiple pairwise comparisons The Kaplan–Meier method was used in GraphPad Prism 8.0 to estimate 5-year PFS and OS as the primary and secondary endpoints, respectively. To assess the importance of potential prognostic factors, univariate analysis was performed using log-rank test and Cox’s proportional hazards regression model. Sample size for each group was presented in the respective figure and table legends. *P* < 0.05 was considered statistically significant.

## Result

### Characteristics of patients

The characteristics of patients in groups 5-Fu and group control are listed in Table 1. There was no significant difference between the two groups in terms of age, gender, clinical stage, and depth of invasion (*P* > 0.05). Therefore, the background data of all patients were relatively similar.

**Table 1:** Baseline clinical characteristics of the patients

		5-FU (*n* = 111)	Control (*n* = 54)	*P*
Age				0.370
≥ 65		31 (27.9%)	19 (35.2%)	
< 65		80 (72.1%)	35 (64.8%)	
Sex				0.237
Male		62 (55.9%)	36 (66.7%)	
Females		49 (44.1%)	18 (33.3%)	
Stage				0.06
1		21 (18.9%)	10 (18.5%)	
2		46 (41.4%)	15 (27.8%)	
3		43 (38.7%)	25 (46.3%)	
4		1 (0.9%)	4 (7.4%)	
T				0.299
1		1 (0.9%)	3 (5.6%)	
2		25 (22.5%)	12 (22.2%)	
3		44 (39.6%)	18 (33.3%)	
4		41 (36.9%)	21 (38.9%)	
N				0.218
0		67 (60.4%)	25 (46.3%)	
1		32 (28.8%)	20 (37.0%)	
2		12 (10.8%)	9 (16.7%)	
Part				0.742
Colon		52 (46.8%)	27 (50.0%)	
Rectal		59 (53.2%)	27 (50.0%)	
Part				0.562
Right		21 (18.9%)	7 (12.9%)	
Left		25 (22.5%)	15 (27.8%)	
Transverse colon		6 (5.4%)	5 (9.3%)	
Rectal		59 (53.2%)	27 (50.0%)	

### Long-term prognosis

The median follow-up for long-term outcomes was 100 months (range 60–120 months). Thirty-two (28.82%) postoperative metastasis occurred in group 1 and twenty-seven (50%) in group 2 (*P* = 0.01); of these, three (2.7%) cases developed postoperative abdominopelvic metastases in group 5-FU and five (9.3%) cases recurred postoperatively in group control. Seven (6.3%) patients in group 5-FU developed liver metastasis and eight (14.8%) patients in group control developed liver metastasis. Eleven (9.9%) cases of pulmonary recurrence occurred in group 5-FU and five (9.3%) cases in group control, without statistically significant differences. Two (1.8%) patients in group 5-Fu developed colon metastasis and three (5.6%) patients in group control developed colon metastasis. Three (2.7%) cases of rectum recurrence occurred in group 5-Fu and two (3.7%) cases in group control. No (0.0%) patients in group 5-Fu developed lymph node metastasis and two (3.7%) patients in group control developed rectum metastasis. Seven (6.3%) cases of other organs recurrence occurred in group 5-Fu and five (9.3%) cases in group control. Among them, in the 5-Fu group, one case simultaneously had metastasis of liver and other organs. Within the control group, one case had simultaneous metastasis of liver, lung, and abdominopelvic. One case had a simultaneous metastasis of the liver and the abdominopelvic cavity. There was no statistical difference in the metastatic sites between the two groups (Table 5), which may be related to too few metastatic cases.

**Fig. 2:**
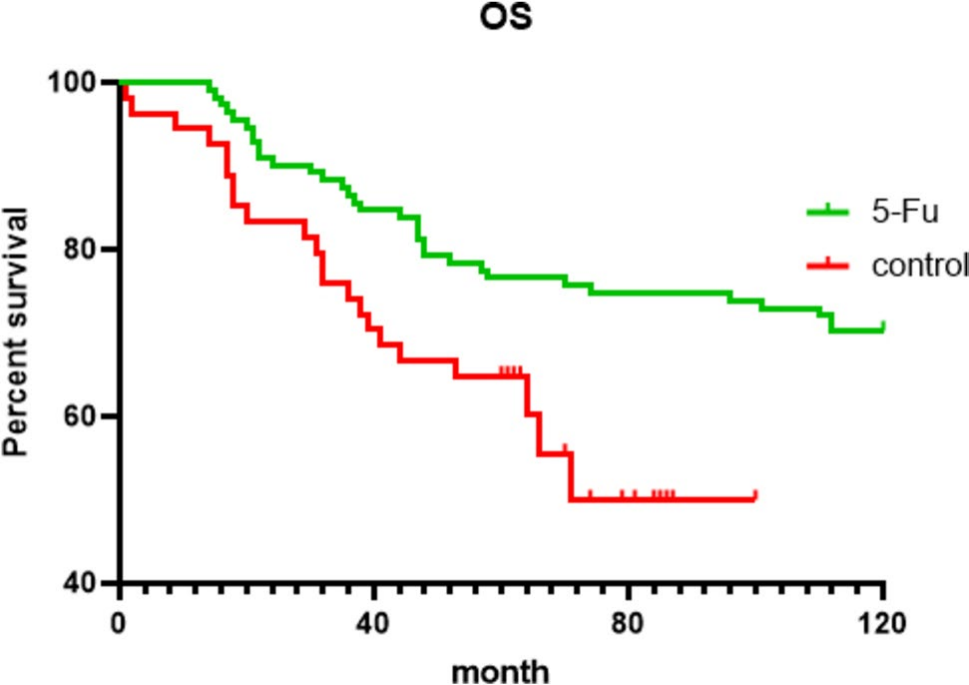
Overall survival (OS) curves (5-FU, green line; control, red line). The group-specific 5-year overall survival rates were 76.58% in 5-FU implants group and 64.81% in control group. These differences were statistically significant (*P* = 0.013)

**Fig. 3:**
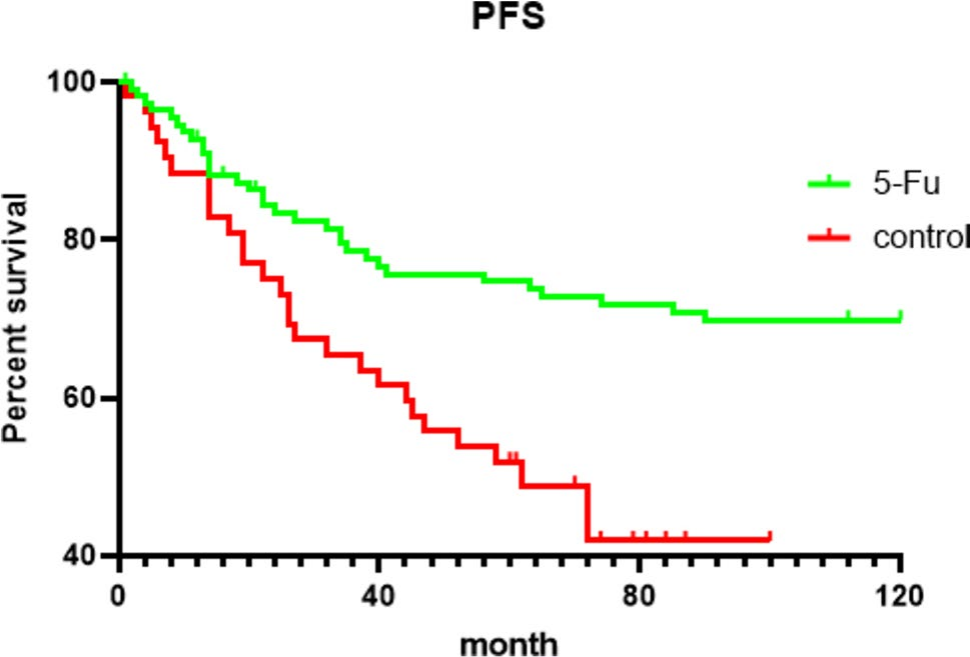
Progression-free survival (PFS) curves (5-FU, green line; control, red line). The 5-year PFS was 75.68% in 5-FU implants group and 53.70% in control group (*P* = 0.002)

We can see in the Figs. 2 and 3, for 5-year OS, there were (111–26) / 111 76.58% for the 5-FU group and (54–19) / 54 64.81% for the control group (*P* = 0.013, Fig. 2); for 5-year PFS, there were (111–27) / 111 75.68% in the 5-FU group and (54–25) / 54 53.70% in the control group (*P* = 0.02, Fig. 3). All of the experimental groups outperformed the control group, and the experimental outcomes varied greatly.

### Short-term prognosis

The incidence of postoperative complications in both groups is shown in Table 2. There was no significant difference between the two groups in terms of postoperative fever, incision infection, gas transit time, and pelvic effusion (*P* > 0.05). Table 2 shows that there were no significant differences between the two groups in terms of hospital stay, postoperative hospital days, hyperthermia (greater than 39.5 °C) as well as the incidence of secondary surgery (see Table 3).

**Table 2:** Postoperative complications, length of hospital stay, and adverse effects in patients

Parameter	5-FU (*n* = 111)	Control (*n* = 54)	*P* value
Hospital stay after operation (days)	10.14 ± 4.298	10.74 ± 4.606	0.415
Time to first flatus (days)	2.68 ± 0.572	2.80 ± 0.595	0.543
First defecation time (days)	4.02 ± 0.751	4.15 ± 0.763	0.422
Peritoneal infection	2	2	0.598
Pelvic effusion	3	1	1.00
Anastomotic leakage	1	1	0.549
Incisional infection	1	0	1.00
Ileus	1	2	0.250
Postoperative hemorrhage	0	0	-
Hyperthermia	6	8	0.07
Diarrhea	0	0	-
Nausea/vomiting	3	3	0.394
Second operation	3	3	0.394

**Table 3:** Changes of biochemical indexes after operation

Biochemical index changes	5-Fu	Control	*P* value
WBC^a^	3.04 ± 3.56	4.77 ± 3.72	6.66E-03
N^b^	3.60 ± 3.40	6.44 ± 4.53	4.12E-05
L^c^	– 0.53 ± 0.70	– 1.24 ± 3.57	4.77E-02
RBC^d^	– 0.30 ± 0.54	0.77 ± 8.67	1.94E-01
Na	– 1.96 ± 12.65	– 2.45 ± 5.50	3.43E-01
K	0.06 ± 0.64	0.20 ± 0.97	7.83E-01
PLT^e^	– 30.61 ± 51.87	– 36.37 ± 71.00	1.94E-01
ALT^f^	– 1.97 ± 28.58	3.58 ± 65.90	6.39E-01
AST^g^	– 0.10 ± 22.30	7.84 ± 66.00	6.39E-01
SCr^h^	– 4.1268 ± 12.75	– 5.05 ± 16.45	4.92E-01
BUN^i^	– 0.16 ± 1.91	0.43 ± 2.87	4.57E-01
alb^j^	– 5.63 ± 5.81	– 6.88 ± 5.72	4.57E-01

^a^White blood cell (WBC)

^b^Neutrophil (N)

^c^Lymphocyte (L)

^d^Red blood cell (RBC)

^e^Platelet (PLT)

^f^Alanine transaminase (ALT)

^g^Aspartate transaminase (AST)

^h^Serum creatinine (SCr)

^i^Blood urea nitrogen (BUN)

^j^Serum albumin (alb)

Cases of anastomotic leakage were found in both groups, and no patients required revision surgery. Cases of intestinal obstruction were identified in both groups, and ileostomy was performed in both groups.

**Fig. 4:**
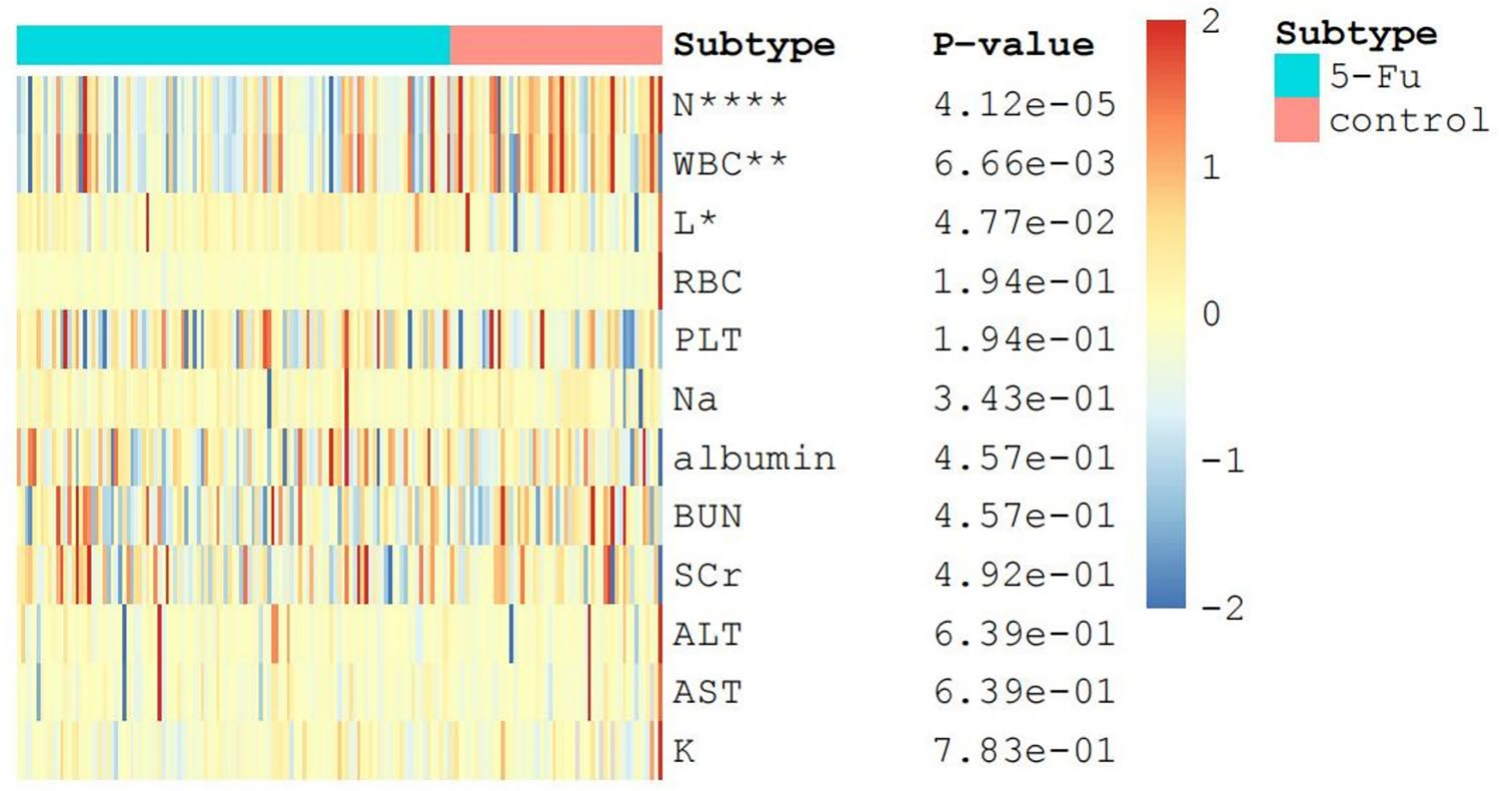
Changes of biochemical indexes after operation

In this paper, as shown in Fig. 4, the differences in the changes of different biochemical indexes between the two groups before and after surgery were also investigated as seen in the graph, there was a significant difference between the two groups regarding the pre- and post-differences in leukocytes, neutrophils, and lymphocytes (*P* < 0.05), while for sodium ions, potassium ions, platelets, alanine transaminase, glutamic oxalacetic transaminase, creatinine, urea as well as albumin, there were no significant differences between the two groups (*P* > 0.05). This may be related to the intraperitoneal chemotherapy implant getting into the circulation.

### Univariate multivariate analysis

The results of the univariate analysis and the Cox model are presented in Table 4. On the basis of univariate analysis, we found that the use of 5-FU implants (*P* < 0.05), depth of invasion (*P* < 0.05), and stage (*P* < 0.05) had an impact on the 5-year OS and PFS rates. Multivariate analysis suggested that 5-FU implants can reduce the risk of 52.6% [95% CI 1.006–3.244; *P* = 0.048] for OS and 46.5% [95% CI 1.211–3.600; *P* = 0.008] for PFS (see Table 5).

**Table 4:** Univariate and multivariate survival analysis of overall survival (OS) and progression-free survival (PFS) predictors

		OS					PFS				
Parameter		Univariate analysis		Multivariate analysis			Univariate analysis		Multivariate analysis		
		Log rank *χ*2	*P* value	HR	95% CI	*P* value	Log rank *χ*2	*P* value	HR	95% CI	*P* value
Age (years)		0.592	0.441				0.155	0.694			
≥ 65	50										
< 65	115										
Sex		0.093	0.761				1.769	0.184			
Males	98										
Females	67										
Stages		14.247	0.003	1.498	0.97–2.314	0.068	14.772	0.02	1.5	0.991–2.271	0.055
I	31										
II	61										
III	68										
IV	5										
Depth of invasion		10.778	0.013				10.354	0.016			
T1	4					0.086					0.164
T2	37			1.996	0.378–10.555	0.416			1.389	0.268–7.193	0.695
T3	62			0.358	0.130–0.989	0.048			0.398	0.153–1.036	0.059
T4	62			0.694	0.391–1.233	0.213			0.709	0.407–1.236	0.226
Lymph node		3.14	0.208				4.453	0.108			
0	92										
1	52										
2	21										
Implants		6.181	0.013	1.806	1.006–3.244	0.048	9.938	0.002	2.088	1.211–3.600	0.008
Non-5-FU implants	111										
5-FU implants	54										
Part		0.14	0.708								
Colon	79										
Rectal	86							0.582	0.446		
Chemotherapy		0.297	0.586					2.619	0.106		
1	118										
0	47

**Table 5:** Recurrence site

Parameter	5-FU (*n* = 111)	Control (*n* = 54)	*P* value
Liver	7 (6.3%)	8 (14.8%)	0.088
Lung	11 (9.9%)	5 (9.3%)	1
Abdominopelvic	3 (2.7%)	5 (9.3%)	0.115
Colon	2 (1.8%)	3 (5.6%)	0.332
Rectum	3 (2.7%)	2 (3.7%)	0.663
Lymph node	0 (0.0%)	2 (3.7%)	0.106
Other organs	7 (6.3%)	5 (9.3%)	0.531

## Discussion

Some studies have shown an overall recurrence rate of 27% and a local recurrence rate of 12% in colorectal cancer (Malcolm et al. 1981; Obrand and Gordon 1997). In general, the local recurrence rates of colon cancer are lower than those of rectal cancer (Harris et al. 2002; Obrand and Gordon 1997; Yun et al. 2008). Cases of recurrent disease after curative surgery for colorectal cancer accounts for approximately 30–50% of cases. The annual rate of recurrent disease after radical resection was approximately 9.9%, 26.2% at 3 years, and 31.5% at 5 years (Guraya 2019). Pugh et al. conducted a randomized controlled trial evaluating patients after CRC and found that 189 (17%) experienced recurrent disease. Researchers have found that the incidence of recurrent disease varied depending on the location of the primary tumor; 14% in the right colon, 16% in the left colon, and 21% in the rectum. Postoperative recurrence was significantly associated with the location of the primary tumor and has a great impact on patient prognosis (Harris et al. 2002). The study concluded that pulmonary recurrences were most often associated with rectal tumors, while multisite recurrences occurred more frequently in right-sided colorectal cancer (Pugh et al. 2016). Similarly, in a retrospective study by Augestad et al. aimed at determining the pattern of metastatic disease after curative surgery for CRC, the authors reported an incidence of recurrent disease in the right colon, left colon, high rectum, and low rectum of 11.2%, 12.8%, 22.2%, and 24.2%, respectively (Augestad et al. 2015).

However, there was no discernible difference in the recurrence rate between the colon and the rectum, as well as across the various locations, in the current investigation. Intraperitoneal recurrence is a common phenomenon in colorectal cancer patients where intraperitoneal spread can be induced by medical means when tumor cells or emboli detach from the dissected lymphatics, intestinal lumen, or reach the peritoneal ducts during surgery (Koppe et al. 2006). Tumor spillage, lymphatic and vascular transection due to surgical opening, or postoperative infection due to anastomotic leak have been shown to be associated with a higher rate of tumor recurrence (Lemoine et al. 2016; Marcuello et al. 2018; Sluiter et al. 2016). Although hematogenous dissemination poses the greatest threat to colorectal cancer patients, peritoneal cancer (PC) also is a relatively common event in patients with recurrent colorectal cancer due to seeding of cancer cells in the peritoneal cavity (Koppe et al. 2006). Peritoneal free cancer cell (pfcc) implantation into the peritoneum is generally accepted as one of the major causes of cancer recurrence (Marutsuka et al. 2003). The common distant metastatic sites of colorectal cancer are the liver and peritoneum. Peritoneal cancer is considered an advanced stage of colorectal cancer, and it is the only site of metastatic disease in 41–45% of patients with metastatic colorectal cancer, suggesting that peritoneal spread may represent a locally advanced colorectal cancer (Pretzsch et al. 2019).

And local recurrence is a key determinant of prognosis in patients with colorectal cancer (Harris et al. 2002). Postoperative local recurrence occurred within 2 and 3 years with a probability of 59.9% and 82.4%, respectively. Recurrences occurred at and around the anastomosis in 45 (49.5%) and pelvic in 28 (30.8%). The overall 5-year survival rate for patients with local recurrence was 28.0%. In patients with local recurrence and no metastasis, the overall 5-year survival rate is 32.8% (Yun et al. 2008). In advanced colon cancer (stage II or III), adjuvant chemotherapy is considered to reduce the rate of local recurrence (Brenner et al. 2014; Harris et al. 2002; Morris et al. 2006; Posner et al. 1993). For rectal cancer patients with advanced rectal cancer (stage II or III) who did not receive chemoradiotherapy preoperatively, the local recurrence rate was lower in patients who received adjuvant chemoradiotherapy postoperatively than in those who received surgery alone, but the difference was not significant (Wein et al. 2000; Yun et al. 2008).

Targeting the potential cause and administering the appropriate medication might, in theory, lower the risk of postoperative recurrence and increase patient survival.

Up to this point, a few other targeted therapies have been developed, including HIPEC, and intraoperative chemotherapy for IOC with varied regimens. Some initial progress have been made with these treatment options.

IOC treatment of CRC is associated with a more favorable survival prognosis. CRC patients treated with IOP survived longer than those who did not. Patients who received both IOC and postoperative chemotherapy (POC) survived longer than those who received only POC (Liu et al. 2019). Cytoreductive surgery and adjuvant therapy HIPEC have been shown to be effective in selected patients and should, therefore, be considered for patients with respectable PC of colorectal origin (Koppe et al. 2006). Intraoperative intraperitoneal chemotherapy improves the prognosis of patients with locally advanced rectal cancer, but also increases the risk of developing after anterior resection of rectal tumors (Wang et al. 2019). There were no serious adverse effects associated with intraoperative chemotherapy, and the risk of surgical complications was not increased (Zhang et al. 2017). Although one patient who received postoperative chemotherapy was found to have chyle leakage, we believe that it may have been a surgical procedure and there was no chyle leakage in the patients we studied (Isik et al. 2015).

Intraperitoneal implantation of 5-fluorouracil is safe for advanced colorectal cancer and reduces the rate of local recurrence and liver metastasis. Long-term efficacy is reliable and improves long-term survival and disease-free survival (Yuan et al. 2015).

Whether the IOC group is superior to the control group depends on the stage of CRC occurrence. For stage II CRC patients, there was no obvious difference between the two groups in terms of disease-free survival (DFS) and overall survival (OS). In contrast, for patients with stage III CRC, the IOC group had a significant advantage in both DFS and OS (Shang et al. 2021).

Extended release fluorouracil after radical surgery and postoperative adjuvant chemotherapy can considerably lower the probability of peritoneal recurrence and lengthen PFS in stage III gastric cancer. There was no obvious increase in the incidence of side effects compared with the control group (Xu et al. 2021).

Therefore, in addition to surgical treatment as well as adjuvant chemotherapy, IOP therapy should be considered for addition to the treatment regimen.

## Conclusion

For colorectal cancer surgery patients, intraperitoneal implantation of slow-release 5-FU drugs, which is a safe and simple procedure, can improve the prognosis of patients with CRC.

## Data Availability

The datasets generated during and/or analyzed during the current study are available from the corresponding author on reasonable request.
